# The Habitual Drunkards Bill

**Published:** 1901-05-25

**Authors:** 


The Hospital

A Journal of

The Medical Sciences and Hospital Administration.

Vol. XXX.—No. 765.] Edited by Sir Henry Burdett, K.C.B., and Solomon C. Smith, M.D., M.R.C.P. [May 25, 1901.

### The Habitual Drunkards Bill.

Whatever may be the ultimate decision of medical science with regard to the moderate use of alcohol, either as a dietetic or as a curative agent, there can be no question, in the mind of any observant or impartial person, with regard either to the enormous amount of evil which arises from its immoderate use, or to the propriety of any well-considered endeavour on the part of the Government or the Legislature to check this evil by the infliction of appropriate penalties. We have seen, now for many years, the continuance of great efforts on the part of total abstainers from alcohol, whether acting as individuals or combined into associations of various names and descriptions, to deal with the manifold consequences of the abuse of strong drink; and it is highly probable that some expectation of benefit from these efforts has rather tended to weaken the hands of statesmen, and to induce them to wait for the consequences of reforms which, so far, have certainly not been brought about. Experience has shown, moreover, that the duration of any alleged "reform" of a drunkard is exceedingly uncertain, whether such reform be effected with or without the assistance of coercion in an establishment from which alcohol is excluded; the fact being, to all appearance, that a certain amount or continuance of excess produces structural degeneration of the nervous centres of a kind to modify or impair both the will and the understanding, and to deprive the victim of the stability of mind necessary for perseverance in good resolutions. There are two forms of habitual drunkenness; the form which has its origin in disease, which is uncontrollable by any effort on the part of its subjects, which justifies their detention in institutions, and would also justify the setting of limits to their power of wasting or of bequeathing their property, and the form which is merely criminal, the determination of a man or woman who is at once weak and wicked to practise a pleasurable indulgence without any regard to its effects upon the rights, the safety, or the prosperity of others. The form induced by disease seems to fall naturally within the province of the physician, while that induced by criminality seems to fall as naturally within the province of the jurist.

On these grounds, and because we cannot entertain much hope that total abstinence, under any of the pleas advanced by its advocates, will ever deal effectively with a social evil of huge dimensions, we gladly welcome the announcement that the Government has determined to take up the Bill introduced into the House of Lords by the Bishop of Winchester, and after having so enlarged its provisions as to render them commensurate with the conditions to be dealt with, to carry it in its amended form to the House of Commons and to pass it into law during the present session of Parliament. The amended Bill rests upon the sound principle that habitual drunkenness is a crime, the committal of which should both be punished, and, also, as far as possible, prevented. The Bill, in its new form, will empower the police to serve notices upon the sellers of intoxicating drinks in any locality, informing them that A.B. has been convicted of being an habitual drunkard, and warning them not to supply him with alcoholic liquor under pain of fines, for first and for subsequent convictions, which appear sufficient to effect the purposes for which they are to be imposed. The habitual drunkard will himself commit an offence in asking to be served; but this is a part of the proposal which will certainly require careful consideration. There must, of course, be imprisonment in default of payment of any fine; and we have had only too much experience of the inefficacy of successive short periods of imprisonment where habitual drunkards are concerned. If, under the new law, the endeavour to commit the offence of drunkenness is to be placed on the same level with its actual commission, the former, as well as the latter, should be held to justify effective and sufficiently prolonged detention to afford a reasonable hope of cure. We should like to see, moreover, especially as regards female offenders, a clause rendering it a penal offence to treat them with drink. Such a person as the late Jane Cakebread, for example, would obviously have been unable to pile up her unexampled record of convictions, unless she had been supplied with means of getting drunk by the gifts of people who desired to amuse themselves with the sight of her degradation. There will, no doubt, be various possibilities of amending the Bill in points of detail when it reaches the committee stage in the House of Commons; but the general principle which it asserts, the principle that habitual drunkenness is a crime, the accessories to which are as deserving of punishment as the principals, will, we hope, be effectively maintained. Another admirable provision is that habitual drunkenness shall entitle either the husband or the wife of the offender to a judicial separation by order of a magistrate. No small part of the misery caused by the offence is directly due to its destructive influence upon the family and upon the home, and nothing can be more important than that this influence should, as far as possible, be arrested. Philanthropy has had its full opportunity with the drunkard, and it is now high time that the law should step in where philanthropy has failed.

